# Retraction Note: Polymerase θ inhibition activates the cGAS-STING pathway and cooperates with immune checkpoint blockade in models of BRCA-deficient cancer

**DOI:** 10.1038/s41467-023-43803-0

**Published:** 2023-12-11

**Authors:** Jeffrey Patterson-Fortin, Heta Jadhav, Constantia Pantelidou, Tin Phan, Carter Grochala, Anita K. Mehta, Jennifer L. Guerriero, Gerburg M. Wulf, Brian M. Wolpin, Ben Z. Stanger, Andrew J. Aguirre, James M. Cleary, Alan D. D’Andrea, Geoffrey I. Shapiro

**Affiliations:** 1 Department of Medical Oncology, Dana-Farber Cancer Institute, Boston, MA USA; 2 Department of Medicine, Brigham and Women’s Hospital and Harvard Medical School, Boston, MA USA; 3 Department of Radiation Oncology, Dana-Farber Cancer Institute and Harvard Medical School, Boston, MA USA; 4 Department of Surgical Oncology and Harvard Medical School, Brigham and Women’s Hospital, Boston, MA USA; 5 Department of Medicine, Division of Hematology-Oncology, Beth Israel Deaconess Medical Center and Harvard Medical School, Boston, MA USA; 6 Hale Family Center for Pancreatic Cancer Research, Dana-Farber Cancer Institute, Boston, MA USA; 7 Department of Medicine, Division of Gastroenterology, Abramson Family Cancer Research Institute, University of Pennsylvania Perelman School of Medicine, Philadelphia, PA USA; 8 Center for DNA Damage and Repair, Dana-Farber Cancer Institute, Boston, MA USA; 9 Present Address: Bayer Pharmaceuticals, Cambridge, MA USA; 10 Present Address: Arpeggio, Boulder, CO USA; 11 Present Address: Sanofi, Cambridge, MA USA

**Keywords:** Targeted therapies, Cancer immunotherapy, Homologous recombination, Genomic instability, Translational research

Retraction to: *Nature Communications* 10.1038/s41467-023-37096-6, published online 13 March 2023

**RETRACTION NOTE**

The Editors retracted this article because of concerns regarding a number of figures presented in this work. These concerns call into question the integrity of the data and the article’s overall scientific soundness. An investigation conducted after its publication discovered several instances of image overlap and identified irregularities in the source data provided by the authors. The Editors therefore no longer have confidence in the integrity of the data in this article.

All authors agree to this retraction.

